# An Intragenic SRF-Dependent Regulatory Motif Directs Cardiac-Specific microRNA-1-1/133a-2 Expression

**DOI:** 10.1371/journal.pone.0075470

**Published:** 2013-09-13

**Authors:** Qi Li, Junli Guo, Xi Lin, Xiangsheng Yang, Yanlin Ma, Guo-Chang Fan, Jiang Chang

**Affiliations:** 1 Hainan Provincial Key Laboratory for Human Reproductive Medicine and Genetic Research, Cardiovascular Disease and Research Institute, Affiliated Hospital of Hainan Medical College, Haikou, Hainan, China; 2 Hainan Provincial Key Laboratory of Tropical Medicine, Hainan Medical College, Haikou, Hainan, China; 3 Department of Pharmacology and Cell Biophysics, University of Cincinnati College of Medicine, Cincinnati, Ohio, United States of America; 4 Texas A&M University Health Science Center, Institute of Biosciences and Technology, Houston, Texas, United States of America; University of Nevada School of Medicine, United States of America

## Abstract

Transcriptional regulation is essential for any gene expression including microRNA expression. MiR-1-1 and miR-133a-2 are essential microRNAs (miRs) involved in cardiac and skeletal muscle development and diseases. Early studies reveal two regulatory enhancers, an upstream and an intragenic, that direct the miR-1-1 and miR-133a-2 transcripts. In this study, we identify a unique serum response factor (SRF) binding motif within the enhancer through bioinformatic approaches. This motif is evolutionarily conserved and is present in a range of organisms from yeast, flies, to humans. We provide evidence to demonstrate that this regulatory motif is SRF-dependent *in vitro* by electrophoretic mobility shift assay, luciferase activity assay, and endogenous chromatin immunoprecipitation assay followed by DNA sequence confirmation, and *in vivo* by transgenic lacZ reporter mouse studies. Importantly, our transgenic mice indicate that this motif is indispensable for the expression of miR1-1/133a-2 in the heart, but not necessary in skeletal muscle, while the enhancer is sufficient for miR1-1/133a-2 gene expression in both tissues. The mutation of the motif alone completely abolishes miR-1-1/133a-2 gene expression in the animal heart, but not in the skeletal muscle. Our findings reveal an additional architecture of regulatory complex directing miR-1-1/133a-1 gene expression, and demonstrate how this intragenic enhancer differentially manages the expression of the two miRs in the heart and skeletal muscle, respectively.

## Introduction

Transcriptional regulation is a pivotal and precise regulatory mechanism that spatially and temporally controls all gene expression including microRNA genes. Transcription factors play central roles in the transcriptional regulation processes, leading to the activation, inhibition, or tissue-specific expression of the target genes. Serum response factor (SRF) is a member of an ancient family of DNA-binding proteins. It contains a highly conserved DNA binding/dimerization domain of 90 amino acids, termed the MADS box [1]. SRF is a key regulator of immediate early gene expression and of terminal muscle differentiation [2,3]. A large number of cardiac, skeletal, and smooth muscle contractile protein genes contain CArG box sequences, an SRF DNA binding site known as serum response element (SRE), in their promoter regions [4,5]. These muscle-specific genes are dependent upon single or multiple CArG boxes as determinants of strong and weak binding sites [3,6,7]. Besides directly binding to the SRE, SRF also serves as a platform to recruite other muscle regulatory proteins, such as NK2 homeobox 5 (NKx2.5), to enhance its transcriptional regulation [7-11].

Analyses of SRF-null mice and null ES cells reveal severe impediments in mesoderm formation, heart formation, and the expression of SRF-regulated genes [12-16]. Cardiac-specific ablation of the murine SRF gene results in embryonic lethality due to cardiac insufficiency during chamber maturation and blockage in the transcription of downstream gene targets [9,11]. Mice lacking SRF expression in skeletal muscle show severe hypoplasia and fail to grow [8]. All of these studies demonstrate that the diversified regulatory effects of SRF on different tissues are highly dependent on the target gene promoters and enhancer regions. Identification of regulatory motifs of SRF provides fundamental information for the target gene manipulation and regulation. In this study, we identify a unique, highly conserved CArG motif located in the intragenic enhancer region of two microRNAs, miR-1-1 and miR-133a-2. The binding of SRF to this SRE site activates transcription of the bicistronic, precursor RNA encoding the *miR-1-1/133a-2* gene. Furthermore, we demonstrate that this new SRE motif is the critical site by directing the expression of the *miR-1-1/133a-2* gene in cardiac but not in skeletal muscle, which reveals a new regulatory element to differentiate miR-1-1/133a-2 expression between cardiac and skeletal muscle at the transcriptional level. Since miR-1-1/133a-2 is a pair of muscle-specific miRNAs with multiple functions in both cardiac and skeletal muscle proliferation and differentiation, our findings provide new information on cardiac-specific regulation of miR-1-1/133a-2 expression.

## Materials and Methods

### Cell Culture, Plasmid Constructs, and Gene Transient Transfection

Mouse myoblast C2C12 cells were cultured in DMEM medium with 10% FBS. SRF and NKx2.5 expression vectors were generated as described previously [17]. All transient transfections were conducted in C2C12 cells (1×10^5^ cells/well in a 12-well plate for a confluent cell layer) using Lipofectamine 2000 in the Opti-MEM reduced serum medium (Invitrogen).

### Electrophoretic mobility shift assay (EMSA)

The ^32^P-labeled 20 bp long DNA duplex containing one SRE from the intragenic enhancer region of the *miR-1-1/133a-2* gene was used as a probe. The positive SRE probe was derived from a skeletal β-actin promoter containing one SRE as described previously [18]. A mutant SRE probe was used to validate the specificity of binding. The probe sequences were as follows (5’ to 3’): SRE, ATGCCCAGATATGGCCACAG/CTGTGGCCATATCTGGGCAT; and mutant SRE, ATGCAAAGATATAACCACAG/CTGTGGTTATATCTTTGCAT. Experiments examining the supershift by the anti-SRF antibody (G20, Santa Cruz, CA) and competition with unlabeled probe were conducted to verify the SRF-DNA interaction. Nuclear extracts were used for each experiment.

### Chromatin immunoprecipitation (ChIP) assay

To define the interaction of SRF with the intragenic SRE regulatory motif *in vivo*, a ChIP assay was performed as described previously [19]. After the SRF expression vector or the SRF specific siRNA (siSRF) (Thermo Scientific Dharmacon RNAi technologies, L-050116-01-0005, Lafayette, CO) was transfected into C2C12 cells for 36 h, cells were gently fixed with formaldehyde and lysed by sonication. The specific SRF-DNA complex was immunoprecipitated using the anti-SRF antibody. The identity of the DNA fragment isolated from the SRF complex was determined by PCR using primers specific for the intragenic regulatory element, which presumably contains the SRF binding site. The PCR primers for the SRE were as follows (5’ at 4965 nucleotide to 3’ at 4770 nucleotide): CCTGTCATGGGGGTTCTATG/CAGGACAGCTGAGAATGCAG. Final PCR product was then sequenced to confirm the presence of the CArG motif (Lone star Labs, TX). The additional four pairs of non-specific PCR primers (NS-P1 to NS-P4) upstream and downstream of the SRE site were as follows (5’ to 3’): NS-P1 (5132~4936), TAGGCAGCTAGGGAAGCAAA/CTCAGCAGCTCCAATCTTCC; NS-P2 (3613~3526), CTGCTTCGTCCAACAGCATA/TGAGGGACTGATGTGTCCAG; NS-P3 (5589~5404), GAGCTGCATCTTACCCCAAC/CTCACAGAAGGCTGGCTACC; NS-P4 (3084~2879), AGGAAGGGCCTCATCAATTT/CCCAAACAGCACCATTTCTT. The quantitative ChIP assay was performed by using the Abcam ChIP Kit (ab500, UK), and the results were normalized with each histone pull-down Ct value.

### Luciferase assay

To determine the functional significance of the CArG regulatory motif, the 4.4 kb intragenic enhancer region of *miR-1-1/133a-2* containing the SRE was subcloned into a pGL3-basic vector expressing a luciferase reporter gene (Promega) by KpnI and SmaI. A similar vector with the mutant SRF binding site (AAAGATATAA) was constructed as a mutant reporter. Generation of the SRF expression vector and transient transfection were described previously^36^. The luciferase assay was conducted 36 h post transfection, and 20 of 60 μl of the lysis supernatant were used for the measurement of luciferase activity by a Monolight 3010 luminometer (Pharmingen). Each sample was measured twice. All results were normalized to β-galactosidase activity (MRX Revelation, DYNEX Technologies, Inc., Chantilly, VA).

### Generation and analysis of transgenic mice

To delineate the SRF-dependent regulatory element within the intragenic enhancer, we made the transgenic mice expressing the *LacZ* reporter gene to test its function *in vivo*. The generation of the transgenic mice was described previously [18]. Briefly, the highly conserved 4.4 kb intragenic enhancer region of *miR-1-1/133a-2* containing the SRE was subcloned by KpnI and SmaI into the pBS-KS-Hsp68-LacZ vector driven by the Hsp68 promoter. The vector was used for a pronuclear microinjection with FVB background, and a parallel process was performed by using the same enhancer with the mutated SRF binding site (AAAGATATAA). The tissue-specificity and expressional levels are dependent on the intragenic regulatory motif. The F_0_ transgenic mice were analyzed at embryonic stage E10.5 and E11.5 by β-galactosidase staining. Positive lacZ blue was used for genotyping purposes. All experiments with animals were approved by the Institutional Animal Care and Use Committee of the Texas A&M Health Science Center–Houston.

### Statistical analysis

Data were expressed as means + the standard errors of the means. All experiments were conducted at least three times. In two-group comparisons, the unpaired, two-tailed student’s *t* test was used (SigmaPlot, version 11.0). A value of *P* < 0.05 was considered statistically significant.

## Results

### Identification of a potential SRF-dependent regulatory motif within the intragenic enhancer of *miR-1-1/133a-2*

**Figure 1:**
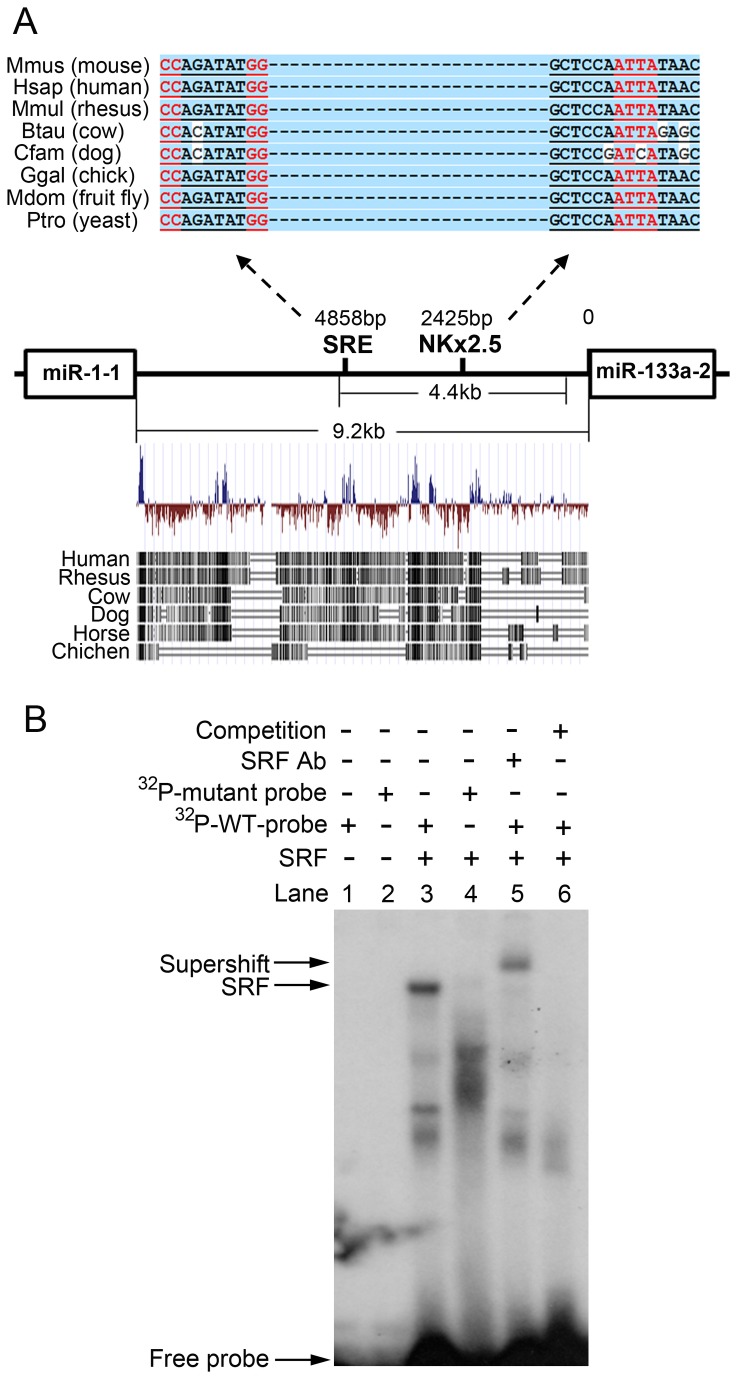
Analysis of *miR-1-1/133a-2* intragenic enhancer region. (**A**) Representative sequence alignments between *miR-1-1* and *miR-133a-2* genes among species. One putative and highly conserved serum response factor (SRF) binding site (SRE) along with one NK2 homeobox 5 (NKx2.5) binding site were suggested by bioinformatic predictions. (**B**) A comprehensive electrophoretic mobility shift assay (EMSA) demonstrated specific interaction between SRF and this SRE motif *in* *vitro*. Ab: antibody. WT: wild-type. Competitor: unlabeled WT probe.

Using the *Silico* database (http://microRNA.sanger.ac.uk), we analyzed 9.2 kb of genomic sequence between the *miR-1-1* and *133a-2* genes, the intragenic enhancer region for the two miRs. We found one potential SRF binding site, SRE (Figure 1A). A comparison of genomic sequences across species revealed that this SRE site was highly conserved among species ranging from yeast to humans. The analysis also showed a conserved NKx2.5 binding site within the intragenic enhancer. Next, we verified the functional role of this perspective motif in the *miR-1-1/133a-2* gene transcription regulation *in vitro* and *in vivo*.

### SRF binds to the CArG motif in the *miR-1-1/133a-2* intragenic enhancer region *in vitro*

To determine whether SRF binds to the identified SRE in the intragenic enhancer region of *miR-1-1/133a-2*, a comprehensive EMSA assay was performed (Figure 1B). A band shift was detected with the addition of the ^32^P-labeled probe containing the perspective SRE and nuclear extract (Figure 1B lane 3), while no SRF binding to the mutant SRE probe was observed, thus validating the binding specificity (Figure 1B lane 4). A supershift band was obtained by adding the anti-SRF antibody (Figure 1B lane 5), confirming the SRF-SRE interaction. Finally, an excess of unlabeled wild-type probe was included in the SRF-SRE binding reaction, which resulted in the abolishment of the band shift (Figure 1B lane 6). This experiment confirmed that SRF was able to bind to the SRE motif in the *miR-1-1/133a-2* intragenic enhancer region *in vitro*.

### SRF interacts with the *miR-1-1/133a-2* intragenic enhancer region *in vivo*

**Figure 2:**
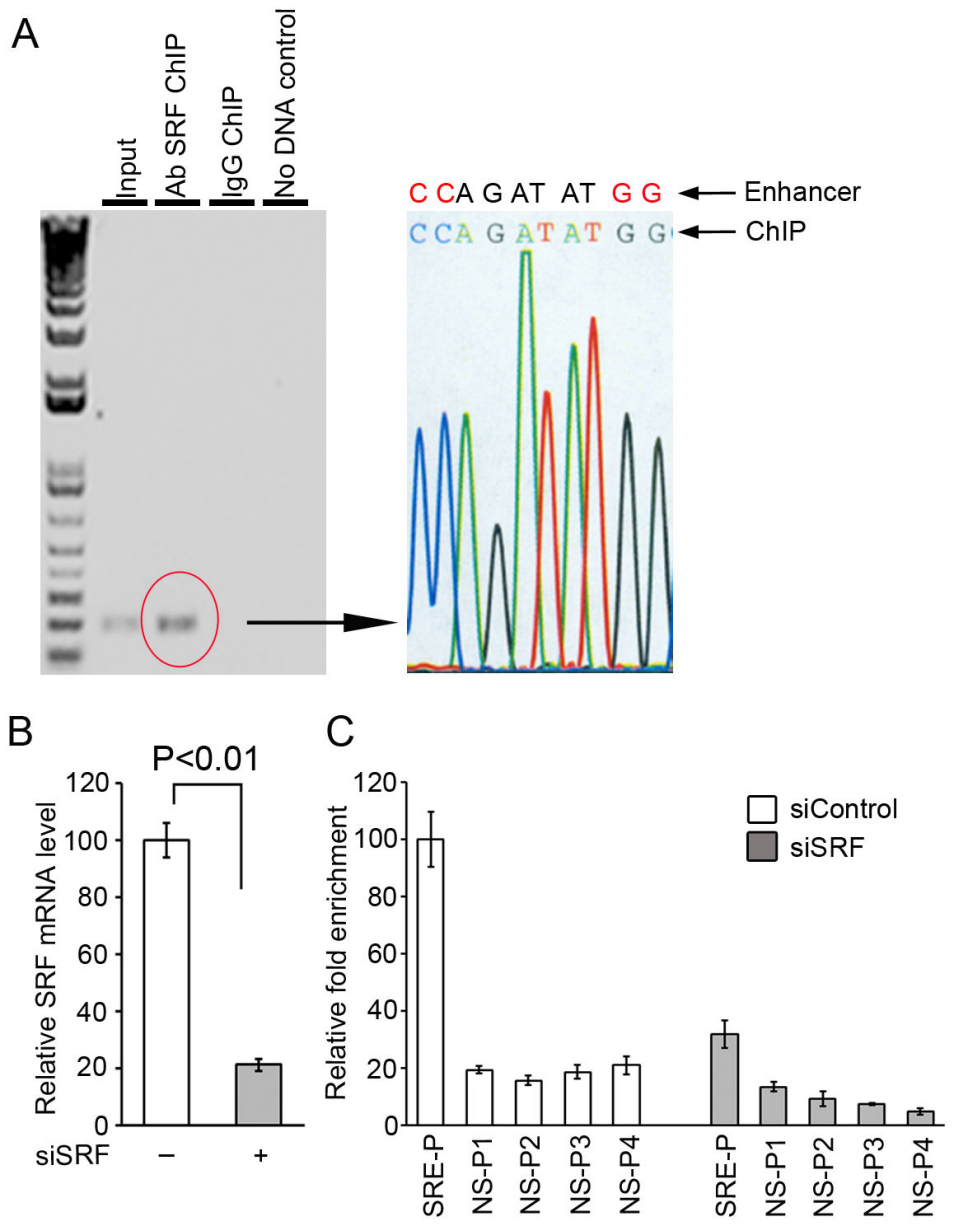
The putative SRE motif located in the intragenic enhancer of *miR-1-1/133a-2* was verified by the chromatin immunoprecipitation (ChIP) assay with sequencing assessment *in vivo*. The cell chromatins were extracted from C2C12 cells transfected with either SRF expression vector or siRNA specific for SRF, and the SRF-SRE complexes were pulled down by the anti-SRF antibody. **A**. The oligonucleotides containing the SRF-binding site were detected (left panel). The PCR product was sequenced and the sequence matched with the enhancer (right panel). **B**. Quantitative PCR showed that SRF knockdown was achieved. **C**. Quantitative ChIP assays showed that loss of SRF resulted in decreases in the SRF-SRE complexes, and non-specific PCR primers failed to detect the SRE motif. SRE-P: specific primers for SRE; NS-P: non-specific primers upstream and downstream of the SRE site.

To verify that SRF interacts with the *miR-1-1/133a-2* intragenic enhancer region through the SRE *in vivo*, a ChIP assay was performed (Figure 2A). The specific SRF-DNA complex was immunoprecipitated using an anti-SRF antibody (Figure 2A left panel). Sequencing analysis revealed that the DNA fragment isolated from the complex with SRF contained part of the *miR-1-1/133a-2* intragenic enhancer sequence with the predicted SRE site (Figure 2 right panel); therefore confirming the interaction of SRF with the CArG box *in vivo* in the *miR-1-1/133a-2* intragenic enhancer region.

To rule out any possible false positive ChIP result and to further verify the specificity of the identified SRE, we conducted two quantitative ChIP assays. The first one was performed when SRF expression was knocked down by siSRF (Figure 2B). The downregulation of SRF resulted in a dramatic decrease in the SRE nucleotides pulled down from the knockdown cells (Figure 2C). The second quantitative ChIP assay was conducted by using four pairs of PCR primers chosen from upstream and downstream of the SRE site. The qPCR results showed that these primers failed to detect the SRE motif. Together, the experiments validated the specificity of the SRE motif and demonstrated the interaction of SRF with the CArG box *in vivo*.

### SRF activates the *miR-1-1/133a-2* intragenic enhancer

**Figure 3:**
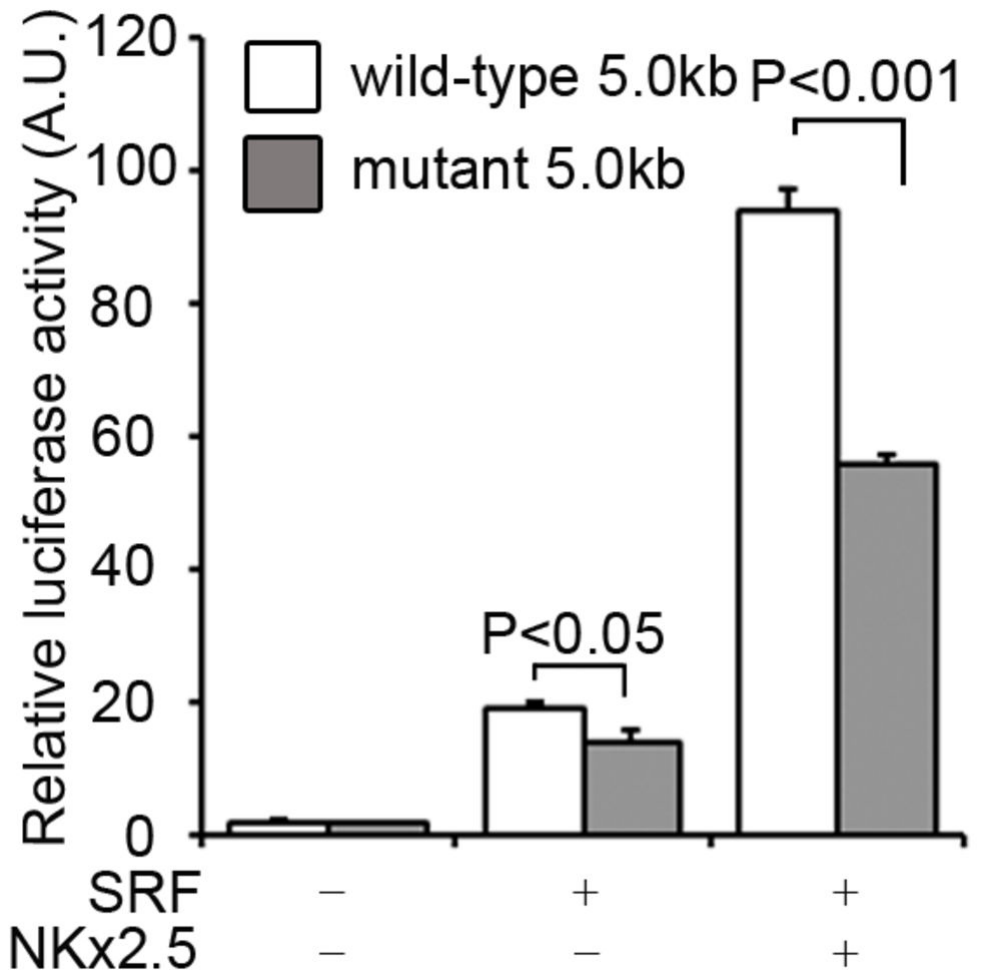
Analysis of *miR-1-1/133a-2* intragenic enhancer region containing the SRE motif by luciferase assay. Empty bar represents reporter vector activity directed by the enhancer with the wild-type SRE motif. Solid bar represents reporter vector activity directed by the enhancer with the mutant SRE motif. SRF increased the luciferase reporter gene activity, and the addition of NKx2.5 facilitated SRF functional activity. Mutation of the SRF binding site resulted in a reduction of the reporter gene activity. The data in each group represents the averages of three experiments with a total of six measurements. Statistical significance was determined by unpaired, two-tailed student’s *t* test. Data are means ± S.E.

To evaluate the functional significance of this SRE, a 4.4-kb-long duplex of the intragenic enhancer containing the SRE was subcloned into a luciferase reporter vector. The chimeric vector can express luciferase as a reporter under *miR-1-1/133a-2* intragenic enhancer control. The effect of SRF on luciferase expression can be detected from the changes in luciferase activity. We found that co-transfection of the reporter and SRF-expressing vectors led to a >18-fold increase in luciferase activity compared to the baseline control without SRF co-expression (Figure 3). In a parallel experiment, the proactive effect of SRF on the *miR-1-1/133a-2* intragenic enhancer was attenuated by the mutation of the SRF binding site, SRE, in the enhancer. Meanwhile, the increased luciferase activity was further enhanced by SRF and NKx2.5 co-transfection, and this enhancement was again lessened by the mutation of the SRE. This experiment indicates that *miR-1-1/133a-2* is regulated by SRF.

The intragenic enhancer directs *miR-1-1/133a-2* gene expression in heart and skeletal muscle, and the SRE in the enhancer is responsible for a differential expression of miR-1-1/133a-2 in the heart but not in skeletal muscle

**Figure 4:**
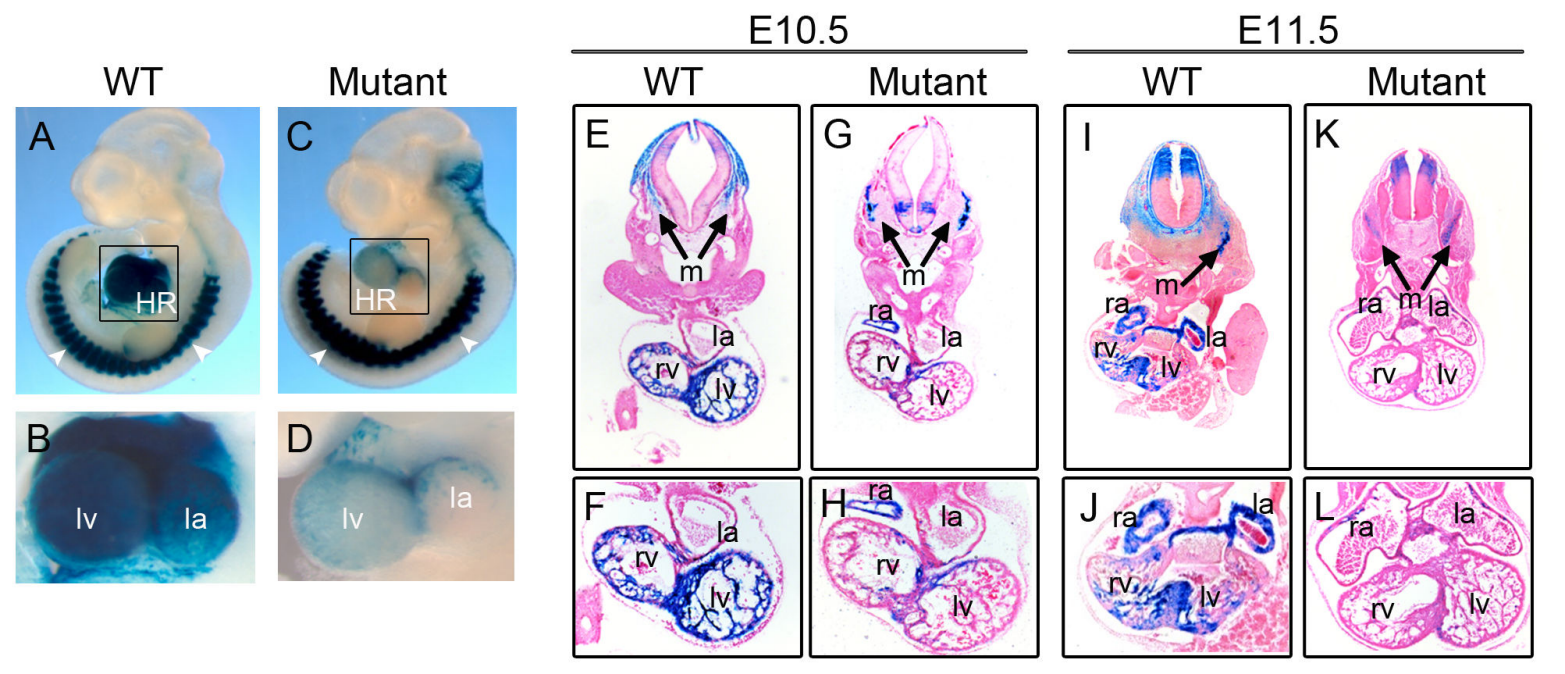
The assessment of *miR-1-1/133a-2* intragenic enhancer using the transgenic mice expressing the reporter gene, *LacZ*, and driven by the intragenic enhancer. β-Galactosidase staining (blue) was performed on embryos (**A**-**D**) and the embryos were sectioned for detailed analysis (**E**-**L**). The enhancer containing wild-type SRE directed the *LacZ* gene expression in heart and somite areas (**A**, **E** and **I**). Close-up of β-Galactosidase staining showed the cardiac-specific expression of lacZ. (**B**, **F** and **J**) The enhancer containing the mutant SRE directed the *LacZ* gene expression in somite areas but not in the heart (**C**, **G** and **K**). Close-up of β-Galactosidase staining displayed the abolishment of lacZ expression in heart (**D**, **H** and **L**). Embryos were at E10.5 (**A**-**H**) and E11.5 (**I**-**L**). HR: heart. lv: left ventricle. rv: right ventricle, la: left atrium. ra: right atrium, m: somite myotomes. White arrows indicate somite myotomes.

Finally, we investigated the role of this SRF-regulated motif in the tissue specificity of miR-1-1/133a-2 expression. Two transgenic mouse lines expressing the reporter gene, *LacZ*, driven by the *miR-1-1/133a-2* enhancer containing the wild-type SRE and the mutant SRE, respectively, were generated. We dissected the mice at two time points, E10.5 and E11.5. We found that the wild-type enhancer was sufficient to direct *LacZ* gene expression in both cardiac and skeletal muscle regions (Figure 4A and 4B). However, the enhancer with the mutant SRE abolished *LacZ* gene expression in the mouse heart but not in skeletal muscle (Figure 4C and 4D). To observe the detailed expression location of the *LacZ* reporter gene, the whole embryos were sectioned. We found that lacZ staining was restricted in cardiac ventricles, atria, and somite myotome areas in wild-type mice (Figure 4E-4F and 4I-4J). The mutation of SRE in the enhancer only diminished *LacZ* reporter expression in cardiac ventricles and atria but not in the somite myotomes (Figure 4G-4H and 4K-4L). This result suggests that the SRF-regulated motif is obligatory for cardiac-specific expression of miR-1-1/133a-2.

## Discussion

MiR-1 and miR-133 are muscle-enriched microRNAs, and they have been demonstrated as critical factors involved in both cardiac and skeletal muscle development and diseases [20-25]. Given that individual microRNAs regulate potentially dozens of genes, functions of miR-1 and miR-133 in cardiac muscle and skeletal muscle can be quite distinct [23,26,27]. Mice with miR-1-2 deletion develop ventricular septal defect (VSD), cardiomyocyte hyperplasia, and abnormal electrophysiology [27]. While the deficiency of miR-133a leads to cardiomyocyte proliferation and VSD. For skeletal muscle, miR-1 facilitates myogenesis, and miR-133 promotes myoblast proliferation [20]. Both miR-1 and miR-133 also participate in cardiomyopathy development including cardiac hypertrophy [25,28], cardiac fibrosis [29,30], and arrhythmia [30,31]. Understanding tissue-specific transcription regulation will provide a foundation for us to manipulate the expression of the two miRs spatially.

There are two copies of miR-1 and miR-133, the miR-1-2/133a-1 and miR-1-1/133a-2. The transcriptional regulation and the enhancer dissection of *miR-1-2/133a-1* have been investigated [32]. In this study, we focused on miR-1-1/133a-2. MiR-1-1/133a-2 are transcribed as bicistronic transcripts on mouse chromosome 2, and they can be transcriptionally regulated by two regulatory loci, which have been dissected by two independent studies [26,32]. One regulatory locus is located upstream of miR-1-1 [26], while the other locus is the 9.2 kb intragenic enhancer located between *MiR-1-1* and *miR133a-2* (Figure 1A) [32]. The upstream and the intragenic enhancers contain multiple *cis*-regulatory elements allowing different transcription factors, including SRF, myocyte enhancer factor 2 (MEF2), and E-box binding factors, to bind. The upstream enhancer study showed that the *MEF2* site is mainly responsible for skeletal muscle expression, while this regulatory locus relies on SRF for cardiac expression [26]. Meanwhile in the analysis of the intragenic enhancer, miR-1-1/133a-2 expression in skeletal muscle is determined by the intact MEF2 motif [32]. In this study, we expand this observation and provide evidence that one evolutionarily conserved SRF binding site in the intragenic enhancer determines the cardiac-specific expression of miR-1-1/133a-2. Genetic deletion of the SRF binding motif eliminates the intragenic enhancer-directed lacZ expression in the heart but not in somites, consistent with the role of the SRF-regulated motif in the upstream enhancer.

The result from our study adds an additional piece of information into this transcriptional complex that drives the expression of two important, muscle-specific microRNAs. In combination with the other two studies, it is intriguing to realize that cells carry two sets of very similar regulatory complexes, which may suggest several biological significances but not a means of waste. First, the redundancy of transcriptional activity may guarantee a faithful completion of transcriptional regulation for any essential genes even under stress conditions. Secondly, an additional enhancer could function as a backup or a reinforcement to secure the expression as well as the expression patterns of critical genes. Finally, multiple transcriptional motifs may potentiate gene regulation to achieve fine-tuning of temporal and spatial controls of gene expression. Given the requisite role of miR-1 and miR-133 in cell survival and development, it is convincing to believe that the redundant transcription complexes directing miR-1 and miR-133a expression are elegantly developed to ensure cells to survive under evolutionary pressure.

Finally, the pathophysiological consequence of the disruption of SRF factor has been demonstrated to contribute to heart failure in both humans and animals. Under stress conditions, SRF can be cleaved into a dominant negative factor that represses its downstream target gene expression, leading to cardiac dysfunction [17]. Given the importance of miR-1 and miR-133 in various cardiomyopathy developments, such as cardiac hypertrophy, understanding the precise control of SRF-mediated microRNA gene regulation in the heart will provide an additional perspective for the treatment of SRF dysfunction-mediated cardiomyopathy.
